# Blood Group Variations in COVID-19 Convalescent Plasma and Regular Blood Donors: A Comparative Analysis in the Serbian Population

**DOI:** 10.3390/microorganisms12050915

**Published:** 2024-04-30

**Authors:** Jasmina Grujić, Zorana Budakov-Obradović, Jelena Klašnja, Radovan Dinić, Vladimir Dolinaj, Alejandro Cabezas-Cruz, Pavle Banović

**Affiliations:** 1 Department of Transfusiology, Faculty of Medicine in Novi Sad, University of Novi Sad, 21000 Novi Sad, Serbia; zorana.budakov-obradovic@mf.uns.ac.rs (Z.B.-O.); jelena.klasnja@mf.uns.ac.rs (J.K.); 2 Blood Transfusion Institute of Vojvodina, 21000 Novi Sad, Serbia; 3 Diagnostics and Laboratory Research Task Force, Balkan Association for Vector-Borne Diseases, 21000 Novi Sad, Serbia; 4 Transfusion Medicine Department, Emergency Center, University Clinical Centre of Serbia, 11000 Belgrade, Serbia; dinicradovan@yahoo.com; 5 Department of Nursing, Faculty of Medicine in Novi Sad, University of Novi Sad, 21000 Novi Sad, Serbia; vladimir.dolinaj@mf.uns.ac.rs; 6 Department of Anesthesia and Intensive Care, Clinical Centre of Vojvodina, 21000 Novi Sad, Serbia; 7 ANSES, INRAE, Ecole Nationale Vétérinaire d’Alfort, UMR BIPAR, Laboratoire de Santé Animale, F-94700 Maisons-Alfort, France; alejandro.cabezas@vet-alfort.fr; 8 Department of Prevention of Rabies and Other Infectious Diseases, Pasteur Institute Novi Sad, 21000 Novi Sad, Serbia; 9 Department of Microbiology with Parasitology and Immunology, Faculty of Medicine in Novi Sad, University of Novi Sad, 21000 Novi Sad, Serbia

**Keywords:** ABO blood group, COVID-19, SARS-CoV-2 infection, convalescent COVID-19 plasma (CCP), blood donor population

## Abstract

This research explores the association between ABO blood groups and susceptibility to SARS-CoV-2 infection, analyzing Convalescent COVID-19 plasma (CCP) donors (*n* = 500) and healthy whole blood donors (BDs) (*n* = 9678) during the pandemic (1 May 2020 to 30 April 2021). A comparison is made with pre-pandemic BDs (*n* = 11,892) from 1 May 2018 to 30 April 2019. Significant differences in blood group distribution are observed, with blood group A individuals being three times more likely to be CCP donors. Conversely, blood groups B, O, and AB are less associated with CCP donation. Notably, blood group O is more prevalent among regular BDs, suggesting potential resistance to SARS-CoV-2 infection. This study underscores variations in blood group distribution during the pandemic compared to pre-pandemic periods. The findings support previous research indicating a link between blood group antigens and viral susceptibility, including SARS-CoV-2. Understanding these associations has implications for public health strategies, with potential for predicting COVID-19 outcomes and transmission patterns. Further research is crucial to explore molecular and immunological mechanisms, providing valuable insights for targeted preventive strategies and personalized healthcare in managing the impact of COVID-19.

## 1. Introduction

Since the discovery of the specific antigen structure and corresponding antibodies of the ABO blood group system, a question has emerged: Is there a link between a blood group and susceptibility/resistance to different diseases? ABO system carbohydrate antigens, in non-soluble form, are located on the cell surface and widely distributed across various cellular types and biomolecules, including red blood cells, platelets, leukocytes, plasma proteins, and cell surface enzymes, among others [1]. Many pathogens use ABO system antigens as receptors to adhere to host cells [2,3]. Accordingly, the changes in specificity of carbohydrate antigens can prevent pathogen adherence and provide protection to the individual [4]. It has been shown that a specific population of individuals has a gene locus that promotes the secretion of ABO antigens in body fluids (i.e., secretors) [5]. In that case, soluble blood group antigens can serve as protective factors by blocking pathogens via competitive binding and consequential inhibition of pathogen adhesion to non-soluble antigens [2,6,7].

Another significant mechanism involves immunity related to the structure of antigen B, which bears similarity to galactose-α-1,3-galactose (α-Gal) [8]. More precisely, the immunodominant sugar in the terminal position of the carbohydrate chain of the B antigen is α-galactose. Humans develop high antibody titers against α-Gal early in life, providing protection against certain infections [9]. Both in vitro and in vivo studies have shown that anti-α-Gal antibodies enhance complement-mediated neutralization of α-Gal-presenting influenza virus, measles virus, Newcastle disease virus, and Type C retrovirus [10,11,12]. Similarly, the interaction between natural anti-α-Gal antibodies and viral envelope antigen B may activate the complement system and potentially neutralize SARS-CoV-2 in infected patients [8].

Coronavirus disease 2019 (COVID-19), caused by severe acute respiratory syndrome-associated coronavirus 2 (SARS-CoV-2), led to a pandemic and a public health crisis. Since the beginning of the COVID-19 pandemic, various experimental therapeutic protocols and/or repurposed drugs have been used with the goal of reducing disease severity and/or mortality. One of the therapeutic protocols used is passive immunotherapy, where convalescent COVID-19 plasma (CCP) donated by recovered persons is administered to individuals with symptomatic COVID-19 infection [13]. Currently, there is no consensus on which blood group if any is associated with the clinical severity of SARS-CoV-2 infection [14]. Different studies on blood and CCP donors have identified that individuals with blood groups A or B have higher chances of developing symptomatic SARS-CoV-2 infection compared to blood group O [15,16,17]. Blood-group-dependent resistance to symptomatic SARS-CoV-2 infection could be used for the construction of a model based on ABO blood group distribution in a specific population that can predict the incidence of COVID-19 and the spreading of SARS-CoV-2.

In this study, we aim to explore the distribution and frequency of different ABO blood groups among CCP and regular BDs during the COVID-19 pandemic and compare it to the distribution among BDs pre-pandemic in Serbia. Additionally, the study seeks to investigate associations between blood group and COVID-19 susceptibility by comparing blood group frequencies in CCP donors, BDs during the pandemic, and BDs pre-pandemic. The study provides actionable insights that can be translated into public health measures, clinical practices, and further research, contributing to our ongoing efforts to combat and manage the impact of COVID-19.

## 2. Donors and Methods

### 2.1. Study Groups

Frequency of different blood groups during the COVID-19 pandemic (i.e., 1 May 2020–30 April 2021) was assessed by analysis of data from CCP donors (*n* = 500) and regular BDs (*n* = 9678). In order to define the distribution of blood groups before the COVID-19 pandemic, we also analyzed data from BDs (*n* = 11,892) who reported to the Blood Transfusion Institute of Vojvodina in the period of 1 May 2018–30 April 2019.

### 2.2. Inclusion and Exclusion Criteria

Complete CCP and blood donors selection process has been described previously [17]. Briefly, the register of individuals who have recovered from COVID-19 was acquired from the Public Health Institute of Vojvodina under the auspices of the national program for CCP donation coordinated by the Serbian Ministry of Health. Persons who have recovered from mild and moderate COVID-19 were contacted by telephone and asked if they want to become CCP donor candidates.

For the purpose of this study, all exclusion criteria used for routine BDs and CCP donors were implemented. Briefly, individuals with systemic diseases including diabetes and cardiovascular diseases, those aged below 18 or above 65 years, or those with hemoglobin concentration <125 g/L (female) and <135 g/L (male) were excluded from the study. A detailed list of exclusion criteria (i.e., contraindications for blood donation) can be found in [18]. The same exclusion criteria applied to CCP donors with additional conditions: age above 60 years, presence of anti-HLA antibodies, and serum protein concentration <60 g/L. According to the guidelines provided by the Blood Transfusion Institute of Serbia, persons who have recovered from severe COVID-19 (treated in the Intensive Care Unit) were not considered for enrollment in the CCP donation program.

All BD and CCP donor candidates underwent screening for hemoglobin levels using the HemoCue Hb 301 System (HemoCue AB, Angelholm, Sweden) (minimum requirement 13.5 g/dL and 12.5 g/dL for males and females, respectively). Candidates who passed hemoglobin screening needed to complete a BD questionnaire and undergo an interview with a doctor and a physical examination. Finally, blood samples were acquired for further hematological, biochemical, molecular, and serological testing (see Section 2.4 and Section 2.5).

**Figure 1:**
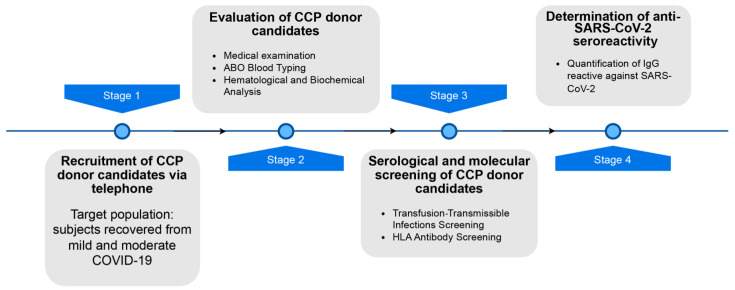
Scheme of CCP donor selection program used during CCP collection campaign. In addition to selection criteria already in place for blood donors, CCP donors needed to (i) be recovered for mild or moderate COVID-19 and (ii) not have HLA antibodies and (iii) have anti-SARS-CoV-2 antibody index > 6. For more information, please see [19]. Scheme created with draw.io.

The CCP donors needed to pass all BD selection criteria and have a previously confirmed SARS-CoV-2 infection, either by real-time reverse transcription polymerase chain reaction from a nasopharyngeal swab specimen or by achieving a satisfactory level of anti-SARS-CoV-2 seroreactivity, regardless of their infection severity. Resolution of symptoms needed to be at least 14 days prior to donation and all CCP donors donated plasma within two months after recovery. They also had to be negative for HLA-specific antibodies, as described previously [19]. The scheme of the CCP donor selection program is outlined in Figure 1.

### 2.3. ABO Blood Typing and Data Access

All BD and CCP donor samples were subjected to ABO blood typing using the fully automated IH-1000 System (Bio-Rad Laboratories, Hercules, CA, USA). ABO blood groups were determined by forward and reverse complete grouping of dipotassium ethylenediaminetetraacetic acid (K2EDTA)-treated venous blood samples via the Gel card technique using DiaClon ABO/D+ Reverse Grouping and ABD-Confirmation assay (Bio-Rad, DiaMed GmbH, Cressier, Switzerland). The tests were performed as per the manufacturer’s instructions.

### 2.4. Hematological and Biochemical Analysis

CCP donors were additionally tested for (i) Complete Blood Count performed on a Celltac Alpha MEK-6500K automated hematology analyzer (Nihon Kohden, Tokyo, Japan); (ii) coagulation status (i.e., prothrombin time, international normalized ratio, and (iii) activated partial thromboplastin time) conducted on a Siemens BCS XP fully automated hemostasis analyzer (Siemens Healthcare GmbH, Erlangen, Germany); (iv) levels of total proteins, albumins, immunoglobulins (isotypes G, M, and A), alanine aminotransferase, aspartate aminotransferase, gamma-glutamyl transferase, total and direct bilirubin, urea, creatinine, and C-reactive protein using spectrophotometry on a Cobas Integra 400 plus fully automated analyzer (Roche Diagnostics International AG, Rotkreuz, Switzerland).

### 2.5. Screening for Transfusion-Transmissible Infections

During the period covered by this study, all donations were tested on an Alinity s analyzer using suitable Alinity s assays (Abbott, North Chicago, IL, USA) for antibodies against Hepatitis C virus (HCV), Treponema pallidum, human immunodeficiency virus (HIV) type 1 and 2, and Hepatitis B virus (HBV) surface antigen, as well as antigens of HIV and HBV. In addition, nucleic acid testing was also performed on donations for detecting RNA of HIV-1, HCV, and HBV using a Cobas 6800 System with Roche Cobas MPX kit (Roche Diagnostics International AG, Rotkreuz, Switzerland).

### 2.6. HLA Antibody Screening

Screening for HLA antibodies was performed on CCP donor candidates who had a past history of pregnancy or previously received transfusion. The test was carried out with the Luminex bead assay (Lifecodes LSA Single Antigen Antibody detection kits (Immucor, Norcross, GA, USA)) following the manufacturer’s instructions. Data were analyzed using the MATCH IT! Antibody software (v1.5).

### 2.7. Statistical Analysis

Standard descriptive values are summarized for every variable. Percentages were used for categorical variables. Continuous variables were represented via mean and 95% confidence intervals. The association between blood group frequency in the case groups (i.e., CCP donors and BDs during pandemic) and control group (i.e., BDs pre-pandemic) were tested by the two-sided Fisher’s exact test and relative risk (RR) measurement. Statistical significance was considered for *p*-values < 0.05. Data analysis was performed using GraphPad Prism v.8.0.1 (GraphPad Software Inc., La Jolla, CA, USA).

## 3. Results

During the period from 1 May 2018 to 30 April 2019 (i.e., the pre-pandemic period), blood donations were accepted from a total of 11,892 donors. Among them, the most common blood groups were A (4646/11,892; 39.07%) and O (4187/11,892; 35.2%) compared to groups B and AB (2121/11,892; 17.83% and 938/11,892; 7.88%, respectively).

A similar trend in blood group distribution was observed among blood donors (BDs) during the COVID-19 pandemic, as well as in CCP donors during the same time period (1 May 2020 to 30 April 2021). The most common blood group was A (3664/9678; 37.85% and 286/500; 57.19%), followed by O (3555/9678; 36.73% and 121/500; 24.2%), B (1752/9678; 18.10% and 66/500; 13.20%), and AB (707/9678; 7.30% and 27/500; 5.4%) among blood and CCP donors, respectively.

**Figure 2:**
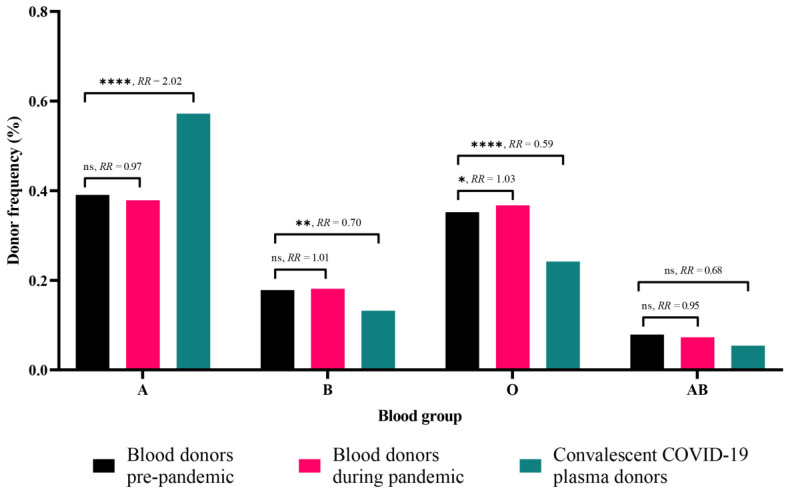
Frequency of blood groups between blood donors and COVID-19 convalescent plasma donors. RR: relative risk, indicates the probability of an individual in the groups of BDs during pandemic and CCP donors to be affiliated to specific blood group within ABO system. The significance of the association was also tested by Fisher’s exact test (* *p* < 0.05; ** *p* < 0.01; **** *p* < 0.0001; ns, non-significant).

Despite similarities in blood group distribution between the case groups (BDs during the pandemic and CCP donors) and the control group (pre-pandemic BDs), Fisher’s exact test revealed a significant difference between pre-pandemic BDs and CCP donors in the frequency of blood groups A (*p* < 0.0001; RR = 2.02), B (*p* = 0.007; RR = 0.70), and O (*p* < 0.0001; RR = 0.59) (see Figure 2). On the other hand, a significant difference was found between pre-pandemic and pandemic BDs only in the frequency of blood group O (*p* < 0.05) (see Figure 2).

When compared with pre-pandemic BDs, the relative risk (RR) analysis revealed that individuals with blood group A were two times more likely to have COVID-19 and be CCP donors. In contrast, individuals with blood groups B, O, and AB were less likely to have COVID-19 and, therefore, less likely to be CCP donors. It is interesting to note that individuals with blood group O were more likely to report as regular BDs, suggesting a possible resistance of this blood group to symptomatic SARS-CoV-2 infection (see Figure 2).

## 4. Discussion

The association between blood groups and susceptibility to viral infection has been a subject of interest even before the COVID-19 pandemic [20]. Previous studies demonstrated that affiliation with specific blood groups within the ABO system exhibits phenotypes and vulnerability to SARS-CoV-1 infection [3,21]. Furthermore, ABO blood group phenotypes have been correlated with the risk of developing COVID-19 [22,23].

Although the underlying mechanism of the association between ABO blood groups and COVID-19 is yet to be determined, hypotheses have been proposed based on previous investigations [15]. Blood group antigens, present on the RBC membrane and in various bodily fluids, may play a role in cellular invasion by viral pathogens, particularly through receptor-mediated affinity [24]. The genetic susceptibility of individual carrying specific blood group-related glycoproteins is thought to be influenced by receptor-mediated affinity, particularly as a means of cellular invasion by viral pathogen. More precisely, ABO blood group antigens are carbohydrates which can be utilized by carbohydrate-binding surface proteins of SARS-CoV-2 and other viruses [25]. Our results support this hypothesis, as we observed differences in blood group distribution among BDs before and during the COVID-19 pandemic, as well as between regular BDs during the pandemic and convalescent CCP donors.

The distribution of the ABO blood group system in Serbia is similar to that in other Balkan countries and Central Europe [26]. In the pre-pandemic period, blood groups A and O were the most common among BDs (39.07% and 35.2%, respectively), while B and AB were less prevalent (17.83% and 7.88%, respectively). When compared with baseline values, we observed a significant difference only in the prevalence of BDs with blood group O, being more represented during the pandemic compared to the pre-pandemic period. No significant difference was found in the frequency of other blood groups in BDs during the pandemic (2020–2021) compared to the pre-pandemic period (2018–2019). It should be highlighted that the number of BDs during the pandemic period decreased by 18% in comparison to the pre-pandemic period. A significant contribution to this phenomenon could be attributed to lockdown measures in Serbia, which ended on 6 May 2020. In addition, it is expected that active COVID-19 infections, as well as distress and fear among BDs, additionally contributed to the decline in blood donations during the pandemic period.

When the ABO blood group distribution within CCP donors (individuals who recovered from COVID-19) was compared to BDs, it was observed that persons with blood group A were two times more likely to report as CCP donors than BDs. In contrast, CCP donors were less likely to have all other blood groups (B, AB, and O) compared to BDs in the pandemic or pre-pandemic period. Given that recent COVID-19 infection was an exclusion criterion for blood donation and inclusion criteria for CCP donation, our results suggest that persons with blood group O are less prone to develop COVID-19 (i.e., more probable to be accepted as BDs), while persons with blood group A are approximately two times more likely to develop symptomatic SARS-CoV-2 infection and to be accepted as CCP donors. The limitation of this study lays in the bias emerging from differences in recruitment between BDs and CCP donors. More precisely, CCP donor recruitment was active (i.e., persons were called by telephone), while BDs were not called by telephone. This bias could have led to a disturbance in the blood group frequencies in the examined sample. In order to address and attenuate identified bias, data from large sample is acquired and analyzed, providing results that should be as representative as possible.

Observed change in ABO blood group frequency in pandemic period may be a consequence of several mechanisms, as demonstrated by the higher likelihood of the SARS-CoV-2 receptor binding domain to bind to A antigens compared to B and H antigens [16]. Additionally, lower angiotensin-converting enzyme-related carboxypeptidase (ACE2) activity in O blood group individuals compared to those with blood group A may provide genetic resistance to SARS-CoV-2 infection [27].

Genetic resistance can also arise from anti-ABO antibodies. After SARS-CoV-2 invades a host cell, the replication process requires the usage of host glycosyltransferases. Consequently, the viral envelope carries a portion of the host cell membrane marked with corresponding blood group carbohydrate antigens specific to the infected individual [28]. Natural antibodies against blood-group-related antigens present on the viral envelope may have a protective role by neutralizing viral particles, blocking them interaction with the ACE2 receptor, and/or eliminating them through opsonization [29].

Guillon et al. demonstrated that natural anti-A antibodies can block S protein binding to ACE-2 receptors, consequently blocking viral invasion of the host cells [30]. The absence of anti-A antibodies may be the reason why individuals with blood group A showed the highest rise in reporting for CCP donation, as they may experience COVID-19 more frequently compared to other blood groups. Conversely, individuals with blood group O carry anti-A and anti-B antibodies, possibly exhibiting resistance to viral particles carrying carbohydrates of groups A and/or B. Significant differences in the levels of anti-A and/or anti-B antibodies were reported between patients with COVID-19 and healthy individuals, indicating that those with low levels of natural anti-A and/or anti-B antibodies would be at a higher risk of SARS-CoV-2 infection [31]. Matzhold et al. observed that individuals with pre-existing high serum concentrations of anti-A/anti-B may have a protective effect against SARS-CoV-2 infection [32]. In addition, the decreased representation of blood group O individuals among CCP donors could be linked to the anti-α-Gal immunity [8]. In addition to anti-A and anti-B, blood group O individuals may exhibit a heightened immune response potentially associated with anti-α-Gal antibodies [33]. The presence of anti-B in blood group O individuals could block SARS-CoV-2 particles from blood group B individuals, while anti-α-Gal antibodies that cross-react with B antigen on the viral surface may contribute to a more robust defense against this pathogen [8]. Interestingly, individuals with B antigens, which do not have significant levels of anti-B and anti-α-Gal, were less represented among CCP donors. Other mechanisms, such as those involving anti-A antibodies described by Guillon et al. [30], could be at play in these individuals in the study population. Only individuals affiliated to the AB blood group have both A and B antigens but are also devoid of both anti-A and anti-B antibodies. As generally observed, individuals observed within the AB blood group were the least represented here, but they were less frequent in the group of CCP donors compared to BDs in the pre-pandemic and pandemic periods. Higher susceptibility to infection for individuals of blood groups A and AB and a lesser risk for blood group O was reported previously [34]. Our results align with these previous observations, as BDs with blood group O were more represented during the pandemic than in the pre-pandemic period, suggesting that these individuals had less frequent COVID-19 or had more common subclinical/abortive SARS-CoV-2 infections that did not evolve into COVID-19. Our findings cannot imply that differences between blood groups affect the absolute susceptibility to infection or protection from infection, since there is a possibility that in certain blood groups the infection goes undetected. In addition, it was demonstrated that non-O blood groups are associated with a higher risk of thrombosis during COVID-19 due to increased levels of von Willebrand factor and Factor VIII [35]. These observations underscore the complex role of blood groups and their associated antibodies in influencing susceptibility to infections, necessitating further research for validation and a comprehensive understanding of these immune interactions. Nevertheless, it should be highlighted that an absence of association between blood types and COVID-19 susceptibility was also reported [36].

## 5. Conclusions

In this study, we investigated the association between ABO blood groups and susceptibility to symptomatic SARS-CoV-2 infection in the Serbian population during the COVID-19 pandemic. Our findings reveal a significant difference in the distribution of blood groups among CCP donors and BDs before and during the pandemic.

Individuals with blood group A showed a threefold higher likelihood of becoming CCP donors, while those with blood groups B, O, and AB were less likely to report for CCP donation. Notably, individuals with blood group O appeared to be more resistant to symptomatic SARS-CoV-2 infection, as they were more likely to remain regular BDs rather than becoming CCP donors.

These results support previous studies suggesting a potential link between blood group antigens and susceptibility/resistance to viral infections, including SARS-CoV-2. The mechanisms behind these associations remain to be fully elucidated, but hypotheses propose that blood group antigens on the cell membrane may influence viral invasion and host immune response. Additionally, the presence of anti-ABO antibodies, particularly anti-A antibodies, may play a protective role by neutralizing viral particles and blocking interactions with host cells.

Understanding the relationship between ABO blood groups and COVID-19 susceptibility has implications for predicting the incidence of COVID-19 and the spread of SARS-CoV-2 in specific populations. Further research is warranted to explore the underlying molecular and immunological mechanisms and to validate these findings in diverse populations. The insights gained from such studies may contribute to the development of targeted preventive strategies and personalized healthcare approaches for managing and mitigating the impact of COVID-19 in the context of immunization against α-Gal [37], which theoretically should provide the protection observed in the cohort affiliated to blood group O.

## Data Availability

Data are contained within the article.
